# MicroRNA miR-188-5p enhances SUMO2/3 conjugation by targeting SENP3 and alleviates focal cerebral ischemia/reperfusion injury in rats

**DOI:** 10.22038/ijbms.2024.76165.16485

**Published:** 2024

**Affiliations:** 1 Department of Anesthesiology, The Affiliated Qingdao Municipal Hospital of Qingdao University, Qingdao, Shandong, China; 2 Department of Anesthesiology, Qingdao Eight People’s Hospital, Qingdao, Shandong, China; 3 Graduate School of Dalian Medical University, Dalian, Liaoning, China

**Keywords:** Conjugated, SUMO2/3, Ischemia/reperfusion injury Ischemic stroke, MiR-188-5p, SENP3

## Abstract

**Objective(s):**

Expression of miR-188-5p changes upon experiencing cerebral I/R injury. SENP3 is a predicted target of miR-188-5p. The study aimed to examine the potential mechanism underlying the miR-188-5p mediated enhancement of SUMO2/3 conjugation via targeting SENP3 and alleviation against cerebral I/R injury.

**Materials and Methods:**

Focal cerebral I/R was established in Sprague–Dawley rats using the MCAO model. The expression of miR-188-5p was modulated through intracerebroventricular (ICV) administration of its mimics or inhibitors. The expression of miR-188-5p, SENP3, and SUMO2/3 was detected using RT-qPCR or western blot analysis. Dual luciferase reporter assays were conducted to demonstrate the targeting effect of miR-188-5p on SENP3 in N2a cells. HE staining and TUNEL staining were performed to evaluate neurocellular morphological changes and detect neurocellular apoptosis, respectively. The extent of neurological deficits was evaluated using mNSS. TTC staining was used to evaluate the infarct area.

**Results:**

In the cerebral ischemic penumbra, the expression of miR-188-5p declined and SENP3 levels increased following I/R. Dual luciferase reporter assays confirmed that miR-188-5p directly acted on SENP3 in N2a cells. As a self-protective mechanism, SUMO2/3 conjugation increased after reperfusion. After ICV administration of miR-188-5p inhibitor, the expression of miR-188-5p was down-regulated, the expression of SENP3 was up-regulated, the SUMO2/3 conjugation decreased, and cerebral I/R injury was exacerbated. However, ICV administration of small hairpin RNA targeting SENP3 partially reversed the effects of the miR-188-5p inhibitor.

**Conclusion:**

MiR-188-5p mitigated cerebral I/R injury by down-regulating SENP3 expression and consequently enhancing SUMO2/3 conjugation in rats.

## Introduction

Stroke often leads to disability and even death. Ischemic stroke accounts for approximately 82% of stroke cases (1-3). Reperfusion injury usually results from blood flow restoration and is known as cerebral ischemia/reperfusion (I/R) injury (4). Cerebral I/R injury ultimately leads to irreversible insults to brain tissue, and therapeutic outcomes remain unsatisfactory (5, 6). Owing to the complex pathophysiology of cerebral I/R injury, it is crucial to completely understand the underlying pathological processes to develop new therapeutic strategies.

MicroRNAs (miRNAs/miRs) are endogenous noncoding RNAs of 21–23 nucleotides in length. They bind to the 3′-untranslated region (3′-UTR) of target genes post-transcriptionally and regulate gene expression (7, 8). Studies have reported that the abundance of some miRNAs dramatically varies upon experiencing cerebral I/R injury, and the regulation of certain miRNA expression effectively influences the degree of nerve injury *in vivo* or *in vitro* (9-11). The stress-related miRNA miR-188-5p influences cell survival, apoptosis, and oxidative stress in various diseases (12). Previous findings (13) have shown that miR-188-5p expression significantly changes upon experiencing cerebral I/R. However, the function of miR-188-5p in the pathological process underlying cerebral I/R remains unclear. Therefore, it is essential to further explore the mechanisms and targets of miR-188-5p in cerebral I/R cases.

Certain substrate proteins can be modified by small ubiquitin-like modifier (SUMO) post-translationally, and the resulting SUMOylated proteins are deSUMOylated by sentrin/SUMO-specific proteases (SENPs) (14, 15). The reversible dynamic processes of SUMOylation and deSUMOylation are involved in various pathological and physiological pathways (16). Extensive SUMO2/3 modification of proteins is considered a protective mechanism against cerebral ischemic stress (17, 18). SUMO-specific protease 3 (SENP3) specifically dissociates SUMO2/3 from substrates (19, 20). Our previous study (21) found that SENP3 expression was elevated during cerebral I/R injury in rats, and down-regulation of SENP3 combined with a mild hypothermia intervention exerted a cerebral protective effect.

Analysis of the TargetScan database (www.targetscan.org) identified a putative binding site between miR-188-5p and 3’-UTR of SENP3. Herein, we confirmed that miR-188-5p targeted SENP3 through methods of interfering with miR-188-5p expression in rats and using dual luciferase reporter assay in N2a cells. We also examined SUMO2/3 conjugation levels, analyzed the extent of cerebral injury, and modulated miR-188-5p and SENP3 expression in the ischemic penumbra. Our data revealed that miR-188-5p exerted a protective effect against cerebral I/R injury in rats by down-regulating SENP3 expression, which consequently enhanced the SUMO2/3 modification rate.

## Materials and Methods

***Experimental animals***

A total of 110 specific-pathogen-free male Sprague-Dawley rats (weighing 240-280 g and aged 6-8 weeks) were provided by Jinan Pengyue Experimental Animal Breeding Co., Ltd (license No. SCXK 20190003). The experiments were approved by the Institutional Research Human or Animal Ethics Committee of Qingdao Municipal Hospital (approval No. 2022083). Experimental procedures were performed in strict adherence to the Health Guide for the Care and Use of Laboratory Animals of United States National Institutes (NIH Publication No. 85-23, revised 1996). Rats were housed in the animal facility of Qingdao Municipal Hospital with free access to water and food. Rats were kept in a room on a 12/12 hr light/dark cycle under the conditions of 24 ^°^C ± 2 ^°^C and 50%-60% humidity.

***Groups and establishment of the focal cerebral I/R injury model***

Rats were randomly divided into the following ten groups: sham group (S group), I/R 6 h group, I/R 24 h group (I/R group), I/R 48 h group, miR-188-5p mimics group (M group), miR-188-5p inhibitor group (I group), miR-188-5p NC group (NC group), SENP3 short hairpin RNA (shRNA) group (sh group), shRNA null group (N group), and miR-188-5p inhibitor+shSENP3 group (I+sh group). Five rats were assigned to each of I/R 6 h, I/R 48 h, M, NC, sh, and N groups. Twenty rats were assigned to each of the other four groups.

After inducing anesthesia by administering an intraperitoneal injection of 30 mg/kg pentobarbital sodium, focal cerebral I/R was established as described previously (22, 23). After isolating the right internal carotid artery (ICA), a filament with a round tip of 0.22 mm diameter was inserted into the middle cerebral artery (MCA) through the right ICA to generate MCA occlusion (MCAO). After 2 hr of blockage, the filament was slowly withdrawn to restore blood flow. To ensure the efficacy of the model, regional cerebral blood flow was measured using a laser Doppler flowmeter (PeriFlux System 5000, Perimed, Stockholm, Sweden). The I/R injury was confirmed when the cerebral blood flow dropped by 85% during MCAO and recovered by 80% after 10 min of reperfusion (24). In the S group, the right ICA was dissected but MCAO was not performed. The mortality rates were as follows: no rats died in the S and I/R 6h groups, one rat (20%) died in each of I/R 48h, M, sh, N, and NC groups, two rats (10%) died in the I/R group, and three rats (15%) died in each of I and I+sh groups. Dead rats were replaced with new rats.

***Intracerebroventricular (ICV) administration of miR-188-5p mimics or inhibitor***

We performed ICV administration of miR-188-5p mimics or inhibitors as described previously (25). After inducing anesthesia through intraperitoneal injection and disinfection, rats underwent stereotaxic surgery for ICV administration using a stereotaxic frame (David Kopf Instruments, Tujunga, CA, USA). The coordinates for ICV injection were 1.0 mm lateral to bregma, 0.5 mm posterior to bregma, and 2.5 mm ventral to the flat skull surface. The rats in the NC, M, and I groups were injected with NC (2.5 μg/2.5 μl), miR-188-5p mimics (2.5 μg/2.5 μl), and miR-188-5p inhibitor (2.5 μg/2.5 μl), respectively, three days before undergoing MCAO. NC, miR-188-5p mimic, and miR-188-5p inhibitor were designed and synthesized by RiboBio (Guangzhou, China).

***Combined ICV of lentivirus of SENP3 shRNA and miR-188-5p inhibitor***

The rats in the I+sh group were injected with lentivirus-containing SENP3 shRNA and miR-188-5p inhibitor as described previously (26). Lentivirus vectors expressing a scrambled shRNA or SENP3 shRNA were designed and purchased from GenePharma (Shanghai, China). A mixture of plasmids comprising pGag/Pol, pRev, pVSV-G (GenePharma), and the constructed plasmid pGLV3/H1+Puro (GenePharma) were cotransfected into HEK-293T cells to generate control lentivirus expressing control shRNAs (lv-null) or shRNAs targeting SENP3 (lv-SENP3). The final titer of the virus (lv-SENP3 and lv-null) was adjusted to 1×10^9^ TU/ml.

Ten microliters of lv-SENP3 or lv-null were injected into the rat cerebral ventricles as described above. Thereafter, rats were administered miR-188-5p inhibitor (four days after lentivirus injection) and subjected to cerebral I/R injury (seven days after lentivirus injection). 

***Dual luciferase reporter assay in N2a cells***

Potential targets of miR-188-5p were identified using TargetScan bioinformatics software. A putative binding site between the 3’-UTR of SENP3 and miR-188-5p was found. We verified whether miR-188-5p directly acted on SENP3 in N2a cells using a dual luciferase reporter assay (27). N2a cells were purchased from Procell Life Science and Technology Co., Ltd. Briefly, cells were seeded in 6-well plates at a density of 2×10^5^ cells/well and incubated at 37 ^°^C for 24 hr. Reporter vectors encoding wild-type (WT-SENP3) or mutant (MUT-SENP3) SENP3 (Promega, Madison, WI, USA) were constructed. Then, miR-188-5p or NC mimics were cotransfected with both kinds of vectors into N2a cells. After 24 hr, luciferase activity was detected using a dual luciferase report assay system (Promega Corporation, USA), following the manufacturer’s protocol.

***Evaluation of neurological deficits***

The extent of neurological deficits was evaluated using the modified neurological severity score (mNSS) as described previously (28). There are four aspects (motor, sensation, balance, and reflex) of the mNSS. The scale ranges from 0 to 18 (0 and 18 indicate no nerve defects and the most severe injury, respectively). After 24 hr of reperfusion, the mNSS of five rats from the S, I/R, M, I, NC, and I+sh groups were measured. After scoring, subsequent experiments were performed. 

***Infarct volume analysis***

Five rats from the S, I/R, I, and I+sh groups were euthanized. The brains were quickly removed and kept at -20 ^°^C for 20 min. The brains were sectioned into five consecutive 2 mm thick coronal slices. To evaluate the infarct area, slices were stained with 1% 2,3,5-triphenyl tetrazolium chloride (TTC; Bioss, Beijing, China) at 37 ^°^C for 30 min. Normal brain tissue was dyed in red, whereas infarcted tissue was pale. Slices were imaged to select the infarct area and analyzed using ImageJ software (NIH, Bethesda, MD, USA). The cerebral infarct volume was expressed as a percentage and calculated as follows (21): infarct volume percentage=(contralateral hemisphere volume-non-infarcted volume in the ipsilateral hemisphere)/contralateral hemisphere volume × 100.

***Hematoxylin-eosin*** ***(H*** ***E) staining***

Five rats from the S, I/R, I, and I+sh groups were randomly selected, anesthetized, and sacrificed. The cerebral ischemic cortex was fixed using transcardiac infusion of 0.9% normal saline and 4% paraformaldehyde. Tissue samples were collected from the cortical ischemic penumbra (the junction of infarcted tissue and normal tissue) for HE staining. Briefly, the tissues were immersed in 10% paraformaldehyde/phosphate-buffered saline (PBS) solution overnight at 4 ^°^C. The tissues were then dehydrated, cleared, embedded in paraffin wax, and cut into 4-μm coronal slices. Slices were stained with Harris hematoxylin for 5 min, incubated in 75% hydrochloric acid ethanol for 30 sec, and then immersed in acidified eosin ethanol for 2 min. Stained slices were observed under a microscope (Olympus, Tokyo, Japan) to evaluate the morphological changes of brain tissue cells.

***Terminal deoxynucleotidyl transferase dUTP nick end labeling (TUNEL) staining***

TUNEL staining was used to detect nerve cell apoptosis (29). Five rats from the S, I/R, I, and I+sh groups were sacrificed by decapitation. Sections from the ischemic penumbra were treated as described for HE staining until embedding in paraffin wax and slicing. Afterward, TUNEL staining was performed following the instructions of the TUNEL kit (Merck, Germany). Five randomly nonoverlapping visual fields from each section were observed under a light microscope at 400× magnification. TUNEL-positive cells, which had brown granules in the nucleus, were considered apoptotic cells. The apoptosis rate was calculated as follows: apoptosis rate (%)=number of apoptotic cells/total number of cells×100.

***Reverse transcription-quantitative polymerase chain reaction (RT-qPCR)***

Five rats from each group were selected randomly after reperfusion and were deep anesthetized. The cortical ischemic penumbra was rapidly collected on ice following decapitation for RT-qPCR and western blot analyses as described previously (21). Total RNA was extracted from the cortical ischemic penumbra using a MiniBEST Universal RNA Extraction Kit (TaKaRa, Dalian, China). The PrimeScript^TM^ RT reagent kit with gDNA Eraser (TaKaRa) was employed to reverse mRNAs into cDNAs. Amplification and analysis of cDNAs by qPCR were conducted using an ABI 7500 fast real-time PCR system (Applied Biosystems TM) and SYBR Premix Ex Taq (Tli RNaseH Plus, TaKaRa). Relative expression was determined using the 2^–^^ΔΔCt^ method and normalization to the levels of the internal control β-actin. The primers used are listed in Table 1.

***Western blot analysis***

A bicinchoninic acid protein assay kit (Beyotime Biotechnology) was used to detect protein concentration in the ischemic penumbra. Proteins (30-50 µg) were loaded per well onto 10% sodium dodecyl sulfate-polyacrylamide gels and separated using electrophoresis. Proteins were then transferred onto a polyvinylidene fluoride membrane. The membrane was blocked using 5% skim milk in PBS containing 0.1% Tween 20 (PBST) at room temperature (25 ^°^C) for 1 hr and was incubated with the following primary antibodies (monoclonal, rabbit, Abcam, Cambridge, UK) overnight at 4 ^°^C: anti-SENP3 (ab124790, 1:1000), anti-SUMO2/3 (ab109005), and anti-β-actin** (**monoclonal mouse, ab8226, 1:2000). After washing, the membrane was incubated with goat antirabbit or antimouse horseradish peroxidase-conjugated secondary antibody (Abcam) for 1 hr at room temperature. After another wash, immunoreactive bands were detected using enhanced chemiluminescence (GE Healthcare Bioscience). Densitometry of the images was performed using ImageJ software (NIH) and normalized to the signal obtained for β-actin. 

***Statistical analysis***

SPSS 22.0 statistical software (IBM Corporation, Armonk, NY, USA) was used for data analysis. All data are presented as mean±standard deviation. One-way analysis of variance (ANOVA) was used to compare different groups, repeated-measure ANOVA was used to compare multiple time points in the same group, and the least significant difference *post hoc* test was performed. *P-*value<0.05 was considered statistically significant.

## Results

***Opposite expression patterns of miR-188-5p and SENP3 during cerebral I/R in rats***

**Figure 1:**
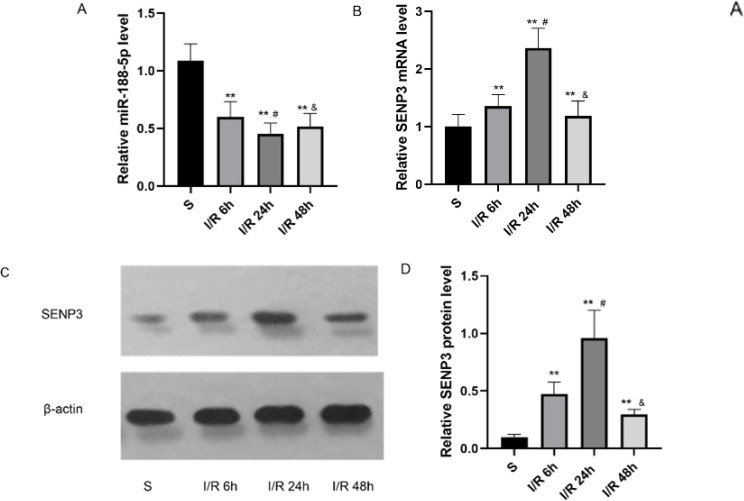
Expression of miR-188-5p and SENP3 in the cortical ischemic penumbra following focal cerebral I/R injury in rats

Expression of miR-188-5p and SENP3 mRNA in the ischemic penumbra was measured using RT-qPCR, and SENP3 protein levels were determined using western blotting 6, 24, and 48 hr after reperfusion. As shown in Figure 1A, the expression of miR-188-5p was reduced at the three time points and was the lowest 24 hr after reperfusion (*P*<0.05). Conversely, SENP3 mRNA (Figure 1B) and protein (Figure 1C, 1D) levels were elevated and peaked 24 hr after reperfusion (*P*<0.05). Since the down-regulation of miR-188-5p expression coincided with higher SENP3 levels, there might be a functional relationship between miR-188-5p and SENP3. As expression changes were maximal 24 hr after reperfusion, we selected this time point for further testing.

***SENP3 is a direct target of miR-188-5p***

**Figure 2:**
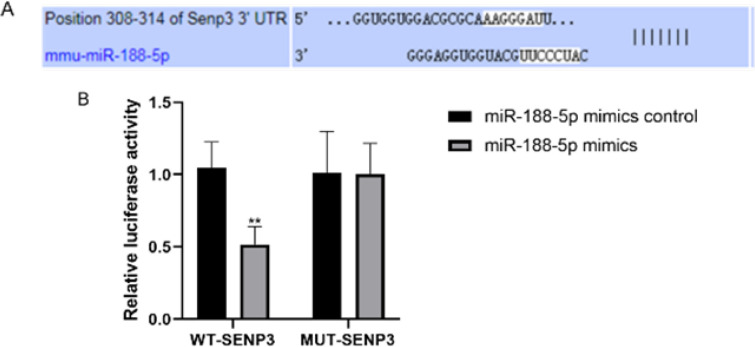
SENP3 is a direct target of miR-188-5p

We used the TargetScan database to predict the targets of miR-188-5p and found that miR-188-5p and SENP3 mRNA had base complementary sequences (Figure 2A). To confirm the association between miRNA-188-5p and SENP3, dual luciferase reporter assays were performed. As presented in Figure 2B, luciferase activity was markedly attenuated in N2a cells cotransfected with miR-188-5p mimics and WT-SENP3 compared with that in cells cotransfected with miR-188-5p mimics and MUT-SENP3 (*P*<0.01). Therefore, miR-188-5p likely regulates the expression of SENP3 by directly binding the 3’-UTR of SENP3.

***Modulating miR-188-5p expression results in changes in SENP3 levels***

**Figure 3:**
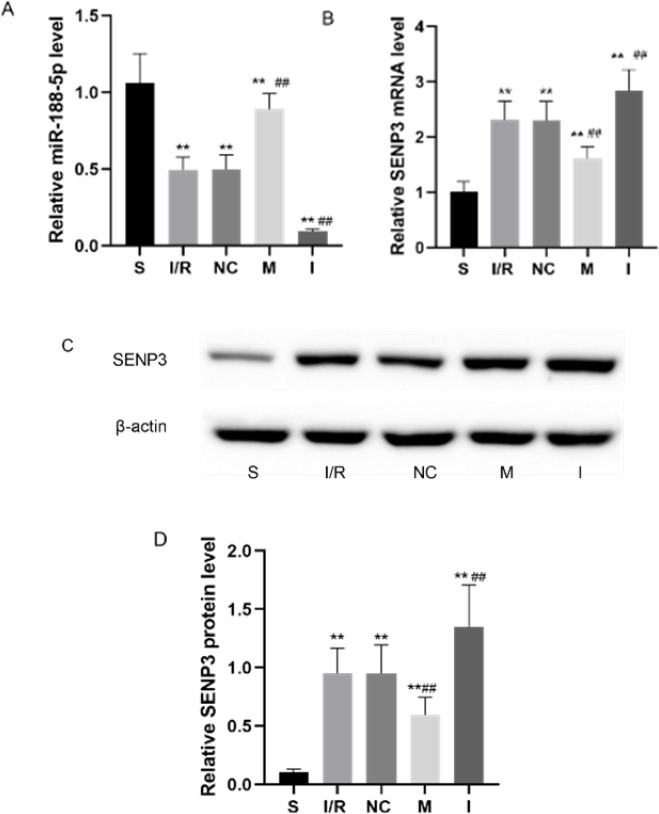
Effects of modulating miR-188-5p expression on SENP3 levels

We modulated the expression of miR-188-5p through ICV administration of miR-188-5p inhibitors or mimics in rats three days before I/R as described previously (25). As shown in Figure 3A, miR-188-5p expression in the cortical ischemic penumbra was examined 24 hr after reperfusion to ensure transfection efficiency. SENP3 levels were also detected in the ischemic penumbra 24 hr after reperfusion. SENP3 mRNA and protein levels declined following the expression of miR-188-5p mimics, whereas the expression of SENP3 increased following injection of miR-188-5p inhibitor (*P*<0.01, Figures 3B, 3C, and 3D). 

***SENP3 shRNA prevents the up-regulation of SENP3 expression induced by miR-188-5p inhibitors in the cerebral I/R rat model***

**Figure 4:**
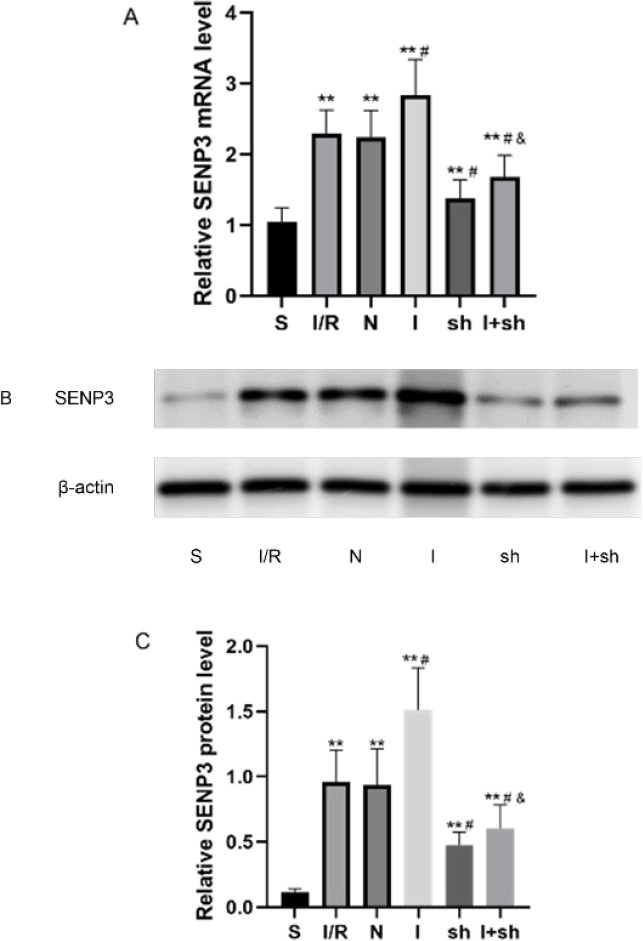
Effects of SENP3 shRNA and miR-188-5p inhibitors on SENP3 expression following focal cerebral I/R injury

A total of 10 μl lv-SENP3 was injected into the cerebral ventricles of rats. According to the instructions, ICV injection of recombinant virus was performed seven days before I/R, and ICV injection of miR-188-5p inhibitor was conducted three days before I/R. As presented in Figure 4, miR-188-5p inhibitor up-regulated the expression of SENP3 24 hr after reperfusion compared with that in the I/R group, whereas SENP3 shRNA down-regulated SENP3 expression (*P*<0.05). Interestingly, combined injections of SENP3 shRNA and miR-188-5p inhibitor resulted in a dramatic down-regulation of the expression of SENP3 compared with that in the I group (*P*<0.05), which indicated that the effect of 188-5p inhibitor on SENP3 was weakened. Further analyses, including the levels of SUMOylation and cerebral injury indicators, were conducted after injection of miR-188-5p inhibitor or of both SENP3 shRNA and miR-188-5p inhibitor.

***SUMO2/3 conjugation to proteins is reduced by miR-188-5p inhibitor during cerebral I/R***

**Figure 5:**
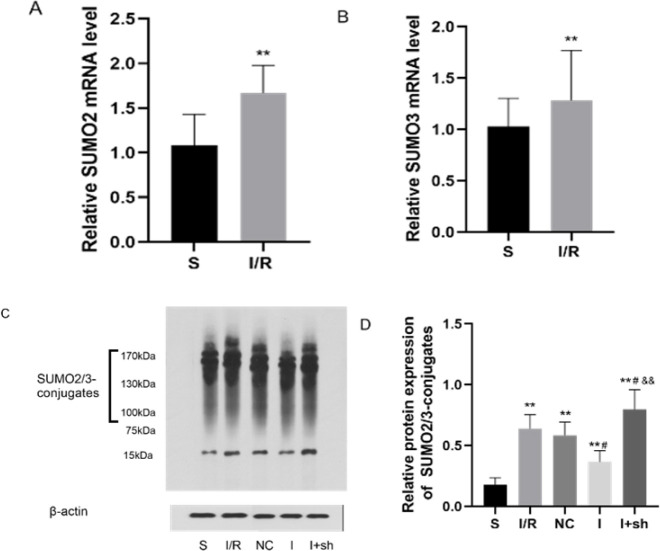
Effects of miR-188-5p inhibitor on SUMO2/3 levels following focal cerebral I/R injury

Expression of SUMO2 and SUMO3 mRNAs in the ischemic penumbra was measured using RT-qPCR, and SUMO2/3 protein levels were determined using western blotting. As described previously (21), a 15-kd band detected on western blots using SUMO2/3-specific antibodies corresponds to free SUMO2/3 proteins, whereas the 130–170-kd signal is conjugated SUMO2/3, representing the degree of SUMO2/3 modification. As presented in Figures 5A and 5B, the expression of SUMO2 and SUMO3 mRNAs increased after reperfusion (*P*<0.01). Figures 5C and 5D show that the expression of conjugated SUMO2/3 was up-regulated in the four groups 24 hr after reperfusion compared with those in the S group (*P*<0.01). Additionally, conjugated SUMO2/3 levels decreased after ICV injection of miR-188-5p inhibitor (*P*<0.05). Remarkably, down-regulation of SENP3 expression reversed the negative effect of miR-188-5p inhibitor on conjugated SUMO2/3 levels (*P*<0.01). Therefore, miR-188-5p likely up-regulates SUMOylation by targeting SENP3. 

***Histopathological changes and apoptosis of neurocytes after reperfusion are aggravated by the miR-188-5p inhibitor***

**Figure 6:**
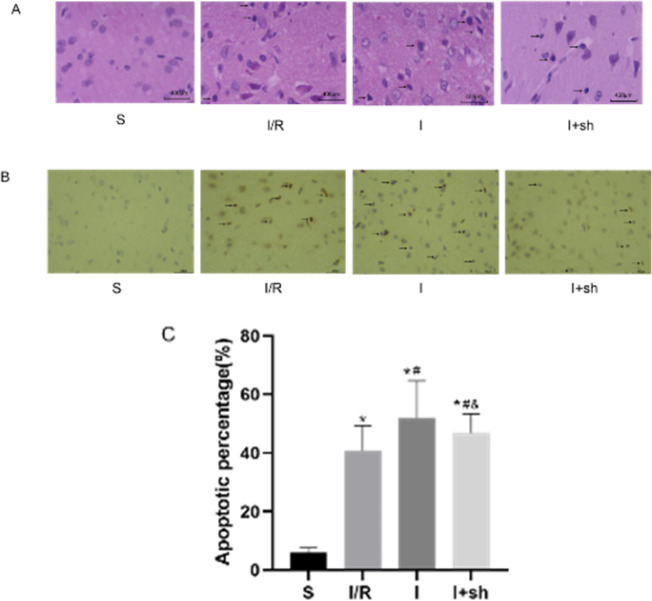
Effects of miR-188-5p inhibitor on histopathological changes and neurocyte apoptosis following reperfusion

Neuronal histopathological changes were examined 24 hr after reperfusion in the ischemic penumbra using HE staining. Neuronal morphology was normal in the S group, whereas shrunken cell bodies and nuclear pyknosis were observed in the other three groups. Injection of miR-188-5p inhibitor further aggravated the neuronal pathological injury following reperfusion. However, SENP3 shRNA attenuated the damaging effects induced by miR-188-5p inhibitor (Figure 6A). 

TUNEL staining was used to detect neuronal cell apoptosis in the ischemic penumbra. Neuronal cell apoptosis rate increased following I/R insult compared with that in the sham group. Moreover, miR-188-5p inhibitor increased the cellular apoptosis rate following reperfusion, whereas SENP3 shRNA partly reversed this stimulatory effect of miR-188-5p inhibitor on apoptosis (*P*<0.05, Figure 6B, 6C).

***SENP3 shRNA reduces the effects of miR-188-5p inhibitor on mNSS and cerebral infarct volume after reperfusion***

**Figure 7:**
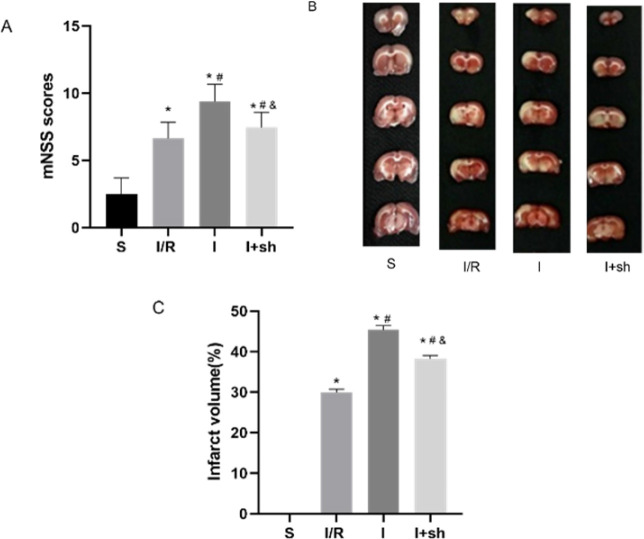
Effects of miR-188-5p inhibitor and SENP3 shRNA on mNSS and cerebral infarct size following reperfusion

Neurological deficits in rats were evaluated using mNSS 24 hr following reperfusion (28). mNSS was significantly increased in the three groups after reperfusion compared with that of the sham group (*P*<0.05). The miR-188-5p inhibitor exacerbated neurological deficits following reperfusion (*P*<0.05). However, SENP3 shRNA attenuated the neurological deficits caused by miR-188-5p inhibitor (*P*<0.05, Figure 7A).

Cerebral infarct volume was determined by performing TTC staining 24 hr after reperfusion. Cerebral infarct volume following reperfusion was further increased by miR-188-5p inhibitor administration (*P*<0.05). Interestingly, SENP3 shRNA attenuated the effect of miR-188-5p inhibitor on the cerebral infarct volume (*P*<0.05, Figure 7B, 7C).

**Table 1:** Primer sequence used in RT-qPCR for each gene or miRNA expression

Gene	Primer sequences
SUMO2	Forward:5′- CGAGAAACCCAAGGAAGGAGTCAAG-3′
	Reverse:5′- AGTCTGCTGCTGGAACACATCAATC-3′
SUMO3	Forward:5′- GATGGCTCGGTGGTACAGTTCAAG-3′
	Reverse:5′- CAATAGCACAGGTCAGGACAACGG-3′
SENP3	Forward:5′- GCACCTCGCTGACATTCCACTG-3′
	Reverse:5′- GGGTCCACCTTAGTCCATCTTCCTC-3′
β-Actin:	Forward:5′- CACCCGCGAGTACAACCTTC -3′
	Reverse:5′- CCCATACCCACCCATCACACC -3′
miR-188-5p	Forward:5′- CATCCCTTGCATGGTGGAG-3′

(A), (B) Expression of miR-188-5p and SENP3 mRNA in the cortical ischemic penumbra assessed by RT-qPCR. (C), (D) Western blot analysis of SENP3 protein levels in the cortical ischemic penumbra. Blots are representative images for the S, I/R 6 h, I/R 24 h, and I/R 48 h groups. (n=5). **P<0.01, versus S group; ^#^*P<*0.05, versus I/R 6 h group; ^&^*P<*0.05, versus I/R 24 h group

(A) Base complementary sequences between miR-188-5p and 3’-UTR of SENP3 obtained using the TargetScan database. (B) Luciferase activity of reporters in N2a cells containing 3’-UTR of WT-SENP3 or MUT-SENP3 after transfection with miR-188-5p mimics or mimics control. (n=5). **P<0.01, versus miR-188-5p mimic control

(A), (B) Expression of miR-188-5p and SENP3 mRNA assessed by RT-qPCR in the S, I/R, NC, I, and M groups. (n=5). **P<0.01 versus S group; ^## ^*P<*0.01 versus I/R group. (C), (D) Western blot analysis of SENP3 protein levels in the ischemic penumbra of the cerebral cortex. (n=5). **P<0.01, versus S group; ^##^*P<*0.01, versus I/R group

(A) Expression of SENP3 mRNA in the S, I/R, N, I, sh, and I+sh groups assessed by RT-qPCR. (B), (C) Western blot analysis of SENP3 protein levels in the ischemic penumbra. (n=5). **P<0.01, versus S group; ^#^*P<*0.05, versus I/R group; ^&^*P<*0.05, versus I group

(A), (B) Expression of SUMO2 and SUMO3 mRNAs in the S and I/R groups as assessed using RT-qPCR. (n = 5). **P<0.01 versus S group. (C), (D) Western blot analysis of SUMO2/3-conjugated protein levels in the ischemic penumbra. (n=5). ^**^P<0.01, versus S group; ^#^ *P<*0.05, versus I/R group; ^&& ^*P<*0.01, versus I group

(A) Representative images of HE staining of the cortical ischemic penumbra. Black arrows indicate cell body shrinkage and nuclear pyknosis, which are typical of cellular apoptosis. (B) Representative images of TUNEL staining of neurocytes. Nuclei with brown/yellow granules (black arrows) are TUNEL-positive cells, which were considered apoptotic. Scale bars: 400 μm. (C) Quantification of the percentage of TUNEL-positive cells. (n=5). *P<0.05, versus S group; ^#^*P*<0.05, versus I/R group; ^&^*P<*0.05, versus I group

(A) Analysis of the mNSS of rats 24 hr after reperfusion. (n=20). (B), (C). The infarct area was detected using TTC staining. (n=5). *P<0.05, versus S group; ^#^*P<*0.05, versus I/R group; ^&^*P<*0.05, versus I group

## Discussion

Early recanalization of cerebrovascular occlusion is an effective treatment of ischemic stroke (30, 31). However, restoration of blood perfusion generally aggravates the initial tissue insult, resulting in cerebral I/R injury (32). The pathological mechanisms of cerebral I/R injury are complex and remain unclear. They include excitotoxicity, oxidative stress, calcium dysregulation, inflammation, necrosis, and apoptosis (33). In this study, reperfusion following MCAO in male rats was used to analyze the pathogenesis of focal cerebral I/R injury. The successful establishment of the model was ensured using transcranial Doppler and mNSS (28). 

Growing evidence has implicated miRNAs in multiple pathological processes involved in cerebrovascular diseases (34, 35). Because they modulate gene expression, miRNAs have emerged as key regulators in cerebral I/R injury (36). It has been reported that more than 20% of miRNA expression changes in the ischemic brain. Therefore, certain miRNAs may play important roles during cerebral ischemia (37, 38). For example, in adult rats, enhanced expression of miR-122 provides neuroprotection against cerebral insults after MCAO (39). Additionally, miR-124 alleviates pyroptosis in rats with cerebral I/R injury (40). Moreover, the sponging action of circCTNNB1 targets miR-96-5p and improves cerebral ischemia injury (41). 

Previous studies have involved miR-188-5p in the mechanism of many types of neoplastic diseases (42-44). Additionally, in myocardial I/R and hypoxia/reoxygenation (H/R) models, miR-188-5p expression is down-regulated, and overexpressing miR-188-5p reduces myocardial I/R or H/R injury (45). Furthermore, miR-188-5p inhibits cellular apoptosis induced by oxygen–glucose deprivation (OGD) in human neural cell lines (12). Consistent with these data, the expression of miR-188-5p decreased 6 hr, 24 hr, and 48 hr after reperfusion in the present study, whereas SENP3 levels were correspondingly increased. Notably, the changes in expression of miR-188-5p and SENP3 were maximal 24 hr after reperfusion. These findings suggested that miR-188-5p participated in the cerebral I/R injury by affecting SENP3 expression.

Subsequently, we confirmed the regulatory relationship between miR-188-5p and SENP3. TargetScan analysis revealed base complementary sequences of miR-188-5p and SENP3 mRNAs. Luciferase reporter assays showed that miR-188-5p directly bound to SENP3 *in vitro*. SENP3 levels were modified *in vivo* by the modulation of the expression of miR-188-5p through ICV injections.

SUMOylation and deSUMOylation are dynamic and reversible and reach an equilibrium that depends on various physiological or pathological processes of the body (16). Several studies have revealed that SUMO modification affects the location and activity of its substrate proteins and regulates pathophysiological processes upon cerebral I/R injury (46-48). Although there are four SUMO proteins (SUMO1, SUMO2, SUMO3, and SUMO4), SUMO2 and SUMO3 are often considered to be SUMO2/3 because 95% of their amino acid sequence are identical (17). SUMO2/3 modification increases in response to stress and reduces stress damage (49). Similarly, increased levels of conjugated SUMO2/3 were observed upon cerebral I/R injury in the present study.

The SENP protease family deconjugates SUMO proteins from substrates. SENP3 specifically catalyzes the deconjugation of SUMO2/3 (50, 51). Interestingly, higher SENP3 expression following reperfusion did not weaken the levels of conjugated SUMO2/3 in the present study. The possible reasons include that (1) as the driving force, the mRNAs expression levels of SUMO2 and SUMO3 increased in the study; (2) the effects of enzymes catalyzing SUMO2/3ylation (e.g., Ubc9) were probably stronger than the effects of deSUMOylating enzymes upon cerebral I/R (52). 

As a self-protective mechanism, SUMO2/3 modification is induced by cerebral ischemic stress to reduce stress damage (53-55). Guo *et al*. (56) found that the deletion of SENP3 prolonged SUMO2/3 modification of Drp1, which alleviated Drp-1-mediated cell death under OGD/reoxygenation. Our previous study also showed that selective intra-arterial brain cooling promoted the occurrence of SUMO2/3 modification, mainly by inhibiting SENP3, and alleviated cerebral I/R injury in rats (21). In the present study, miR-188-5p inhibitor further stimulated the expression of SENP3, resulting in a decline of conjugated SUMO2/3 levels after reperfusion. This coincided with aggravated cerebral insults as evidenced by increased apoptotic rate and cerebral infarct volume, more drastic morphological changes, and higher mNSS. However, rescue experiments performed by injecting SENP3 shRNA mitigated the deleterious consequences of the miR-188-5p inhibitor as conjugated SUMO2/3 levels increased, whereas cerebral injury was less extensive.

Several limitations of this study require further investigation. The expression of Ubc9 should be modulated during cerebral I/R injury to determine the role of SUMOylation. In addition, the protective effects of miR-188-5p up-regulation during cerebral I/R should be assessed. Since miR-188-5p has various possible target genes, other pathways or mechanisms activated by miR-188-5p upon cerebral I/R injury also require further investigation. 

## Conclusion

miR-188-5p alleviates cerebral I/R injury in rats by down-regulating SENP3 expression and consequently enhancing SUMO2/3 conjugation. These findings suggest that inhibition of SENP3 expression through up-regulating miR-188-5p is a promising therapeutic mechanism against cerebral I/R injury.

## Data Availability

The raw data supporting the conclusions of this article are available from the corresponding author upon reasonable request.

## References

[1] Xie W, Zhou P, Sun Y, Meng X, Dai Z, Sun G (2018). Protective effects and target network analysis of ginsenoside rg1 in cerebral ischemia and reperfusion injury: A comprehensive overview of experimental studies. Cells.

[2] Wang YJ, Li ZX, Gu HQ, Zhai Y, Jiang Y, Zhao XQ (2020). China stroke statistics 2019: A report from the national center for healthcare quality management in neurological diseases, china national clinical research center for neurological diseases, the chinese stroke association, national center for chronic and non-communicable disease control and prevention, chinese center for disease control and prevention and institute for global neuroscience and stroke collaborations. Stroke Vasc Neurol.

[3] Cuartero MI, de la Parra J, García-Culebras A, Ballesteros I, Lizasoain I, Moro M (2016). The kynurenine pathway in the acute and chronic phases of cerebral ischemia. Curr Pharm Des.

[4] Liu K, Li L, Liu Z, Li G, Wu Y, Jiang X (2022). Acute administration of metformin protects against neuronal apoptosis induced by cerebral ischemia-reperfusion injury via regulation of the AMPK/CREB/BDNF pathway. Front Pharmacol.

[5] Catanese L, Tarsia J, Fisher M (2017). Acute ischemic stroke therapy overview. Circ Res.

[6] Song L, Mu L, Wang H (2022). MicroRNA-489-3p aggravates neuronal apoptosis and oxidative stress after cerebral ischemia-reperfusion injury. Bioengineered.

[7] Laffont B, Rayner KJ (2017). MicroRNAs in the pathobiology and therapy of atherosclerosis. Can J Cardiol.

[8] Di M, Zhang Y, Zeng R, Liu X, Chen W, Zhang M (2021). The pro-angiogenesis effect of miR33a-5p/Ets-1/DKK1 signaling in ox-LDL induced HUVECs. Int J Biol Sci.

[9] Shi F, Dong Z, Li H, Liu X, Liu H, Dong R (2017). MicroRNA-137 protects neurons against ischemia/reperfusion injury through regulation of the Notch signaling pathway. Exp Cell Res.

[10] Wang N, Zhang L, Lu Y, Zhang M, Zhang Z, Wang K (2017). Down-regulation of microRNA-142-5p attenuates oxygen-glucose deprivation and reoxygenation-induced neuron injury through up-regulating Nrf2/ARE signaling pathway. Biomed Pharmacother.

[11] Wang P, Liang X, Lu Y, Zhao X, Liang J (2016). MicroRNA-93 downregulation ameliorates cerebral ischemic injury through the Nrf2/HO-1 defense pathway. Neurochem Res.

[12] Li L, Cui P, Ge H, Shi Y, Wu X, Fan Ru Z (2020). miR-188-5p inhibits apoptosis of neuronal cells during oxygen-glucose deprivation (OGD)-induced stroke by suppressing PTEN. Exp Mol Pathol.

[13] Gusar VA, Timofeeva AV, Zhanin IS, Shram SI, Pinelis VG (2017). Estimation of time-dependent microrna expression patterns in brain tissue, leukocytes, and blood plasma of rats under photochemically induced focal cerebral ischemia. Mol Biol (Mosk).

[14] Chang HM, Yeh ETH (2020). SUMO: From bench to bedside. Physiol Rev.

[15] Eifler K, Vertegaal AC (2015). Mapping the SUMOylated landscape. FEBS J.

[16] Ovaa H, Vertegaal ACO (2018). Probing ubiquitin and SUMO conjugation and deconjugation. Biochem Soc Trans.

[17] Zhang H, Huang D, Zhou J, Yue Y, Wang X (2019). SUMOylation participates in induction of ischemic tolerance in mice. Brain Res Bull.

[18] Lee YJ, Mou Y, Klimanis D, Bernstock JD, Hallenbeck JM (2014). Global SUMOylation is a molecular mechanism underlying hypothermia-induced ischemic tolerance. Front Cell Neurosci.

[19] Kunz K, Piller T, Müller S (2018). SUMO-specific proteases and isopeptidases of the SENP family at a glance. J Cell Sci.

[20] Guo C, Wilkinson KA, Evans AJ, Rubin PP, Henley JM (2017). SENP3-mediated deSUMOylation of Drp1 facilitates interaction with Mff to promote cell death. Sci Rep.

[21] Sun G, Qin W, Wang Q, Sun X, Chen H, Li J (2021). Selective-cerebral-hypothermia-induced neuroprotection against-focal cerebral ischemia/reperfusion injury is associated with an increase in SUMO2/3 conjugation. Brain Res.

[22] Yang GY, Zhao YJ, Davidson BL, Betz AL (1997). Overexpression of interleukin-1 receptor antagonist in the mouse brain reduces ischemic brain injury. Brain Res.

[23] Belayev L, Alonso OF, Busto R, Zhao W, Ginsberg MD (1996). Middle cerebral artery occlusion in the rat by intraluminal suture Neurological and pathological evaluation of an improved model. Stroke.

[24] Zhao N, Xu X, Jiang Y, Gao J, Wang F, Xu X (2019). Lipocalin-2 may produce damaging effect after cerebral ischemia by inducing astrocytes classical activation. J Neuroinflammation.

[25] Helmschrodt C, Höbel S, Schöniger S, Bauer A, Bonicelli J, Gringmuth M (2017). Polyethylenimine nanoparticle-mediated siRNA delivery to reduce α-synuclein expression in a model of parkinson’s disease. Mol Ther Nucleic Acids.

[26] Yang YQ, Li H, Zhang XS, Li W, Huang LT, Yu Z (2015). Inhibition of SENP3 by lentivirus induces suppression of apoptosis in experimental subarachnoid hemorrhage in rats. Brain Res.

[27] Wei B, Wang Z, Lian Q, Chi B, Ma S (2022). hsa_circ_0139402 promotes bladder cancer progression by regulating hsa-miR-326/PAX8 signaling. Dis Markers.

[28] Li Z, Yang M, Lin Y, Liang S, Liu W, Chen B (2021). Electroacupuncture promotes motor function and functional connectivity in rats with ischemic stroke: An animal resting-state functional magnetic resonance imaging study. Acupunct Med.

[29] Ma B, Liu Y, Zhang X, Zhang R, Zhang Z, Zhang Z (2022). TSPO ligands protect against neuronal damage mediated by LPS-induced BV-2 microglia activation. Oxid Med Cell Longev.

[30] Tsivgoulis G, Saqqur M, Sharma VK, Brunser A, Eggers J, Mikulik R (2020). Timing of recanalization and functional recovery in acute ischemic stroke. J Stroke.

[31] Katan M, Luft A (2018). Global burden of stroke. Semin Neurol.

[32] Jean WC, Spellman SR, Nussbaum ES, Low WC (1998). Reperfusion injury after focal cerebral ischemia: the role of inflammation and the therapeutic horizon. Neurosurgery.

[33] Moskowitz MA, Lo EH, Iadecola C (2010). The science of stroke: Mechanisms in search of treatments. Neuron.

[34] Eyileten C, Wicik Z, De Rosa S, Mirowska-Guzel D, Soplinska A, Indolfi C (2018). MicroRNAs as diagnostic and prognostic biomarkers in ischemic stroke-A comprehensive review and bioinformatic analysis. Cells.

[35] Xu SY, Jiang XL, Liu Q, Xu J, Huang J, Gan SW (2019). Role of rno-miR-124-3p in regulating MCT1 expression in rat brain after permanent focal cerebral ischemia. Genes Dis.

[36] Neag MA, Mitre AO, Burlacu CC, Inceu AI, Mihu C, Melincovici CS (2022). miRNA involvement in cerebral ischemia-reperfusion injury. Front Neurosci.

[37] Min XL, Wang TY, Cao Y, Liu J, Li JT, Wang TH (2015). MicroRNAs: a novel promising therapeutic target for cerebral ischemia/reperfusion injury?. Neural Regen Res.

[38] Kim T, Mehta SL, Morris-Blanco KC, Chokkalla AK, Chelluboina B, Lopez M (2018). The microRNA miR-7a-5p ameliorates ischemic brain damage by repressing α-synuclein. Sci Signal.

[39] Liu da Z, Jickling GC, Ander BP, Hull H, Zhan X, Cox C (2016). Elevating microRNA-122 in blood improves outcomes after temporary middle cerebral artery occlusion in rats. J Cereb Blood Flow Metab.

[40] Sun H, Li JJ, Feng ZR, Liu HY, Meng AG (2020). MicroRNA-124 regulates cell pyroptosis during cerebral ischemia-reperfusion injury by regulating STAT3. Exp Ther Med.

[41] Chen C, Chang X, Zhang S, Zhao Q, Lei C (2022). CircRNA CTNNB1 (circCTNNB1) ameliorates cerebral ischemia/reperfusion injury by sponging miR-96-5p to up-regulate scavenger receptor class B type 1 (SRB1) expression. Bioengineered.

[42] Peng Y, Shen X, Jiang H, Chen Z, Wu J, Zhu Y (2018). miR-188-5p suppresses gastric cancer cell proliferation and invasion via targeting ZFP91. Oncol Res.

[43] Wang M, Zhang H, Yang F, Qiu R, Zhao X, Gong Z (2020). miR-188-5p suppresses cellular proliferation and migration via IL6ST: A potential noninvasive diagnostic biomarker for breast cancer. J Cell Physiol.

[44] Yang X, Wang P (2019). MiR-188-5p and MiR-141-3p influence prognosis of bladder cancer and promote bladder cancer synergistically. Pathol Res Pract.

[45] Xu J, Yu D, Bai X, Zhang P (2021). Long non-coding RNA growth arrest specific transcript 5 acting as a sponge of MicroRNA-188-5p to regulate SMAD family member 2 expression promotes myocardial ischemia-reperfusion injury. Bioengineered.

[46] Choi SG, Kim H, Jeong EI, Lee HJ, Park S, Lee SY (2017). SUMO-Modified FADD recruits cytosolic drp1 and caspase-10 to mitochondria for regulated necrosis. Mol Cell Biol.

[47] Han ZJ, Feng YH, Gu BH, Li YM, Chen H (2018). The post-translational modification, SUMOylation, and cancer (Review). Int J Oncol.

[48] Sun M, Chen X, Yin YX, Gao Y, Zhang L, Chen B (2020). Role of pericyte-derived SENP1 in neuronal injury after brain ischemia. CNS Neurosci Ther.

[49] Brandsma CA, Guryev V, Timens W, Ciconelle A, Postma DS, Bischoff R (2020). Integrated proteogenomic approach identifying a protein signature of COPD and a new splice variant of SORBS1. Thorax.

[50] Martins WC, Tasca CI, Cimarosti H (2016). Battling alzheimer’s disease: Targeting SUMOylation-mediated pathways. Neurochem Res.

[51] Liu K, Guo C, Lao Y, Yang J, Chen F, Zhao Y (2020). A fine-tuning mechanism underlying self-control for autophagy: deSUMOylation of BECN1 by SENP3. Autophagy.

[52] Lee YJ, Miyake S, Wakita H, McMullen DC, Azuma Y, Auh S (2007). Protein SUMOylation is massively increased in hibernation torpor and is critical for the cytoprotection provided by ischemic preconditioning and hypothermia in SHSY5Y cells. J Cereb Blood Flow Metab.

[53] Peters M, Wielsch B, Boltze J (2017). The role of SUMOylation in cerebral hypoxia and ischemia. Neurochem Int.

[54] Hendriks IA, Lyon D, Su D, Skotte NH, Daniel JA, Jensen LJ (2018). Site-specific characterization of endogenous SUMOylation across species and organs. Nat Commun.

[55] Yu S, Galeffi F, Rodriguiz RM, Wang Z, Shen Y, Lyu J (2020). Small ubiquitin-like modifier 2 (SUMO2) is critical for memory processes in mice. FASEB J.

[56] Guo C, Hildick KL, Luo J, Dearden L, Wilkinson KA, Henley JM (2013). SENP3-mediated deSUMOylation of dynamin-related protein 1 promotes cell death following ischaemia. EMBO J.

